# Polyurethane Impregnation for Improving the Mechanical and the Water Resistance of Polypropylene-Based Textiles

**DOI:** 10.3390/ma14081951

**Published:** 2021-04-13

**Authors:** Antonella Patti, Francesco Costa, Marta Perrotti, Domenico Barbarino, Domenico Acierno

**Affiliations:** 1 Department of Civil Engineering and Architecture (DICAr), University of Catania, Viale Andrea Doria 6, 95125 Catania, Italy; 2 Kuvera Spa, Interporto di Nola-Lotto H, 80035 Naples, Italy; f.costa@carpisa.com (F.C.); marta.perrotti@carpisa.com (M.P.); domenico.barbarino@carpisa.com (D.B.); 3 CRdC Nuove Tecnologie per le Attività Produttive Scarl, Via Nuova Agnano 11, 80125 Naples, Italy

**Keywords:** impregnation, polyurethane waterborne dispersion, breaking load, tear strength, abrasion, water resistance

## Abstract

Commercial waterborne polyurethane (PU) dispersions, different in chemistry and selected on the basis of eco-friendly components, have been applied to a common polypropylene (PP)-based woven fabric. Impregnation has been chosen as a textile treatment for improving the features of basic technical textiles in light of potential applicability in luggage and bag production. The effect of drying method, performed under conditions achieved by varying the process temperature and pressure, on the features of the treated textiles, has been verified. The prepared specimens were characterized in terms of mechanical behavior (tensile, tear and abrasion resistance) and water resistance (surface wettability and hydrostatic pressure throughout the treated textiles). The experimental results suggest an incremental improvement of the tensile features for all the investigated specimens. For tear strength, no augmentation compared to that of the neat textile, could be verified as a consequence of polyurethane treatment. Remarkable improvements of abrasion resistance were displayed for all the impregnated PP textiles. Benefits in water resistance could be attributed to the presence of hydrophobic PU in the textile weaving of the PP samples. The ultimate improvement in water resistance was dependent on drying conditions.

## 1. Introduction

The main synthetic fibers involved in the production of technical textiles are made from polyamide (i.e., nylon, PA), polyester (i.e., polyethylene terephthalate, PET), or polyolefins (i.e., polypropylene, PP). Polypropylene fibers offer low cost, ease of manipulation in filament formation, acceptable tensile features, chemical and microbial inertness, and ready recyclability. On the basis of the specific gravity, PP fibers (0.90 g/cm^3^) are lightweight compared to those of PET (1.38 g/cm^3^) and PA (1.14 g/cm^3^): this implies more filaments, constituting a fabric with denser texture, for an equal weight, and at lower cost per unit volume [1]. PP fibers display good fatigue resistance in comparison with other fibers. [2] The melting point of polypropylene, approximately in the range of 160–170 °C, is lower than those of the nylon and polyester, thereby requiring use of lower temperatures, yet this value is sufficient for several textile applications allowing the use of PP fabrics in the production of carpet and geotextile membranes, in filtration and protective clothing, in ropes and upholstery, in laundry and dye bags, in candlewicks and knitted outerwear [3].

From industrial applications, through various technical uses, to the usual indoor or outdoor actions of everyday life, synthetic or natural fabrics must withstand severe working conditions. These may require exposure to tension or abrasion forces, contact with water, oils or chemicals that reduce the functionality and alter the durability over time. In order to improve the overall performance of common fabrics, the application of a polymer to the textile surface has been investigated [4,5]. The treatment of PP nonwoven textiles with water-based acrylic copolymer emulsions, has been examined. Application is as manual screen, followed by drying at 60–70 °C, with fixation carried out at 135 °C for 5 min. Tensile, tear and abrasion testing demonstrated good breaking load and abrasion resistance, and a worsening of the tear strength compared to the same properties of the starting material [6]. A recent investigation of the abrasion characteristics and air and water permeability of PP-coated fabrics has been conducted. A potential application was windbreaker material for sportswear. As treatment, a paste made of aliphatic PU, both polyether–or polyester-based, crosslinker and thickener, was applied using a laboratory coating machine, and then cured at temperatures of 120 or 140 °C. The coating did not improve the waterproofness to desired levels (1300 mm water column hydrostatic pressure) but provided enhanced abrasion resistance, and water vapor and air permeability. After washing, the appearance of the fabric coated with polyether PU-based coating seemed to be better than that of the fabric coated with the polyester-based PU [7].

**Figure 1:**
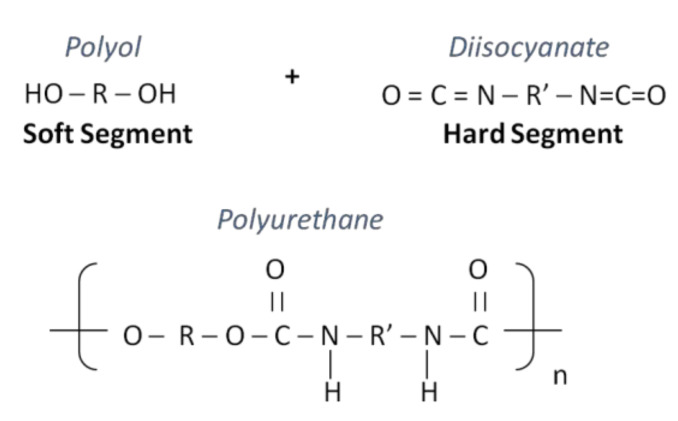
Polyurethane chemical structure.

The PU macromolecule is comprised of alternating non-polar units derived from an isocyanate (aliphatic or aromatic), referred to as the hard segment (HS), and a non-polar macrodiol-derived portion, mainly polyester, polyether–and polycarbonate-based, called the soft segment (SS) (Figure 1). Each of the segments provides the polymer with specific characteristics. In particular, the HS is considered responsible of the stiffness, the film-forming ability and the resistance to the abrasion of the polymer; on the other hand, the SS contributes to elastomeric properties [8]. The chemistry of these portions plays an important role in affecting the final features of PU-based materials. For example, in the HS portion, aliphatic isocyanates offer a better environmental stability compared to aromatic-based formulations [9]. For the SS unit, the polyester-based polyols to provide a high breaking load and outstanding features in terms of resistance against light and ageing. Polyether-based polyols provide chain elasticity and flexibility, but are negatively impacted by heat, light and oxygen. PUs from polyols based on polycarbonate species showed good hydrolysis and aging resistance, and satisfactory mechanical strength [10,11].

Polyurethane waterborne dispersions (PUDs) are a new widespread class of binder systems [12], intended to meet the requirements of environmental sustainability and eco-friendly technologies, by reducing the quantities of toxic and polluting solvents, the emissions of volatile organic compounds (VOC) and the release of the free isocyanates into the atmosphere [13]. Recently, PUDs have found applications in fields such as insulating resources [14], water-based inks [15], potential biomaterials [16], adhesives and coatings [17], leather surface finishing [18] or textiles [19]. The wide range of uses of PUD-based materials is due to the versatility of pristine constituents [20] by which elastomeric, thermosetting or thermoplastic behavior can be obtained [16]. Generally, a PU polymer is hydrophobic in nature and insoluble in water. For improving dispersion in aqueous liquids, the inclusion of specific chemical groups into PU backbones is required. Depending on the character of these chemical moieties, three types of dispersions can be distinguished: (i) anionic by sulfonic, phosphonate or carboxyl groups; (ii) cationic by nitrogen-containing alkyl diols or with sulfur-containing diols; iii) non-ionic realized through zwitterionomers [13]. Many other novel species have been investigated to synthesize aqueous polyurethanes for different purposes [21,22]. Water, oil or strain resistance has been obtained through durable water repellent (DWR) species, i.e., polymeric per–and polyfluoroalkyl substances (PFASs) attached to a non-fluorinated polymeric backbone. Due to the environmental impact of long-chain PFASs (i.e., alkyl chain containing six or more carbons), also coupled with dangerous effects on human health, short-chain PFASs (i.e., waxes and silicones) have been proposed [23].

**Figure 2:**
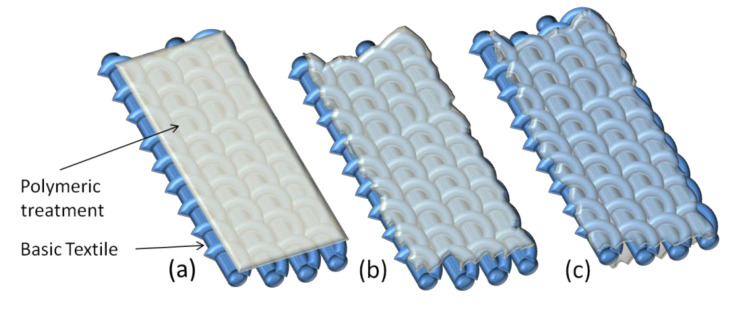
Different appearance of common treated textiles by lamination (**a**), coating (**b**), or impregnation (**c**).

In this work, four types of commercial PUD emulsions (polyether-, polyester-, polycarbonate-based polyols, and incorporating short-chains of polyfluoroalkyl species), were adopted as treatment for improving the overall characteristics of common polypropylene fabrics. A potential application of final products was materials in semi-rigid luggage production. As a PUD application method, the fabric impregnation was preferred to the most known coating and lamination technologies (Figure 2), for preserving the aesthetics of pristine textile and avoiding an excessive increase of weight. The PU polymer was distributed on yarns and filaments of the textile weaving to act as protection for the overall structure.

Testing of mechanical performances, analyzed in terms of tensile, tear and abrasion strength, and water resistance, evaluated through the measurement of surface wettability and maximum allowable hydrostatic pressure thought out the thickness, have been performed on impregnated fabrics. To propose a practical solution, reproducible on industrial scale, with low process times and low energy consumption, PU dispersions, able to form film at ambient conditions, have been chosen. The drying phase was carried out by setting different process parameters (temperature and pressure) and evaluating the processing time and performance of prepared materials.

## 2. Materials and Methods

### 2.1. Materials

A commercial plain polypropylene-based woven fabric (100% PP), with a nominal mass per unit of area equal to 280 g/m^2^ was used in the following experimentation. Three different types of anionic waterborne dispersions were kindly supplied by ICAP SIRA-Chemicals and Polymers Spa (Milan, Italy): a medium soft-aliphatic polyurethane binder polyether-based one (CLEANCAP 800 A), a medium-rigid aliphatic polyester-based polyurethane one (IDROCAP 983PF), and a high-rigid aliphatic polyurethane polycarbonate-based one (IDROCAP 993 PF). PUD based on a perfluoropolyether backbone (FLUOROLINK P56) was a kind gift of Solvay (Bruxelles, Belgium). The technical data of all chosen impregnating products are reported in Table 1.

### 2.2. Sample Preparation

**Figure 3:**
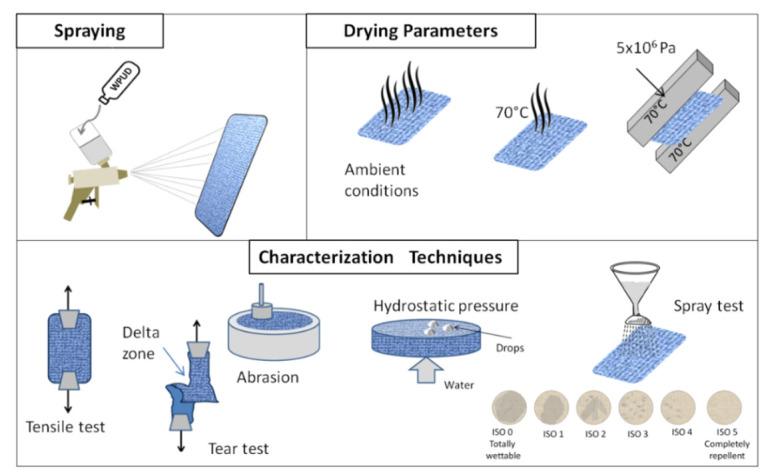
Sample preparation and characterization techniques.

A nominal amount of dispersion was applied to both sides of square piece of fabric (25 × 25 cm^2^) through a spray gun (W-400 ANEST IWATA Corporation, Kyoto, Japan). All impregnated specimens were prepared so as to keep constant the nominal PU weight applied to the textile (7 g of polymer per 625 cm^2^ area of fabric). Preliminary tests were conducted so to determine the minimum amount of dispersion for wetting the entire sample area. Soaked samples were dried at room conditions (T = 25 °C, RH = 50%), in a climatic chamber (mod. 250 E, Angelantoni Industrie spa, Perugia, Italy). When required, the drying operation was performed in a lab oven (mod. Binder, by Geass SrL, Torino, Italy) at temperature of 70 °C, or in a hydraulic press (mod. LP420B, produced by LabTech Engineering Company Ltd, Samut Prakarn, Thailand), at 70 °C of temperature and 5 × 10^6^ Pa of pressure (see Figure 3). Samples were periodically weighed until the measurement has reached a constant value. In all cases, before testing, the specimens remained in controlled conditions (T = 25 ± 2 °C, RH = 50 ± 4%).

### 2.3. Characterization Techniques

#### 2.3.1. Measurement of Mass Per Unit of Area

Mass per unit of area has been calculated for final samples, as described in the standard EN 12127, from the ratio between fabric weight and the corresponding area (20 × 20 cm^2^).

#### 2.3.2. Measurement of Tensile Breaking Load

A universal testing machine (mod. Tensometer 2020) produced by Alpha Technologies INSTRON (Norwood, Massachusetts, USA), equipped with a load cell of 5 kN, was used for measuring the tensile properties of PUD impregnated samples. Tests were performed according to EN ISO 13934-2 (grab method). Both ends of specimens (200 mm in height, 100 mm in width) were fixed in the middle to the clamps of tensometer (size of 25 × 40 mm^2^). A gauge length of 100 mm was ensured. The crossheader was moved with a speed of 50 mm/min, and stopped when a large amount of yarns both in the weft and warp direction was broken. During experiments, load-displacement curves were recorded by the Tensile 2020 software. The breaking load was intended the force at which a great number of yarns was broken in weft and warp direction. For reproducibility at least five specimens were tested.

#### 2.3.3. Measurement of Tear Strength

Tear features were determined on dynamometer equipped with a load cell of 500 N in accordance with the ASTM D2261 standard. Samples were prepared by cutting a piece of fabric 200 mm in length and 75 mm in width. An incision of 75 mm was made along the longitudinal axis of symmetry of the sample. Two tongues were produced by the opening of sample cutting and fixed in the jaws. During testing, the crosshead was set in motion with a rate of 50 mm/min, and was stopped after an elongation of 70 mm. At the end of test, the tear strength was calculated by an average of the five highest peaks on the load-displacement curve. A minimum number of five specimens were considered for reproducibility.

#### 2.3.4. Measurement of Abrasion Resistance

The Martindale tester used in this investigation, was manufactured by C&B Tessile SrL (Milan, Italy), according to standard EN ISO 12947. Specimens were prepared by a ring-shaped cutter (38 mm in diameter) and attached to a circular holding base. A load of 12 kN was applied to them. During testing, they were rubbed with translational movements against a reference abrading textile. The abrasion resistance was determined by the number of cycles for which the breaking of each sample occurred.

#### 2.3.5. Measurement of Waterproofness

The hydrostatic pressure was measured by using a hydrostatic head tester, produced by C&B Tessile SrL, in compliance with standard EN ISO 20811. A piece of fabric (square shape, size of 250 × 250 mm^2^) was firmly clamped on the upper part of a pressure chamber so to ensure negligible leaking of water from locking flanges. A constantly increasing water pressure was exerted from the bottom. The test finished when the water crossed throughout the sample and appeared in three different points on the upper surface. The final pressure was considered the maximum value for fabric waterproofness, and was expressed in millimeters of conventional water column (mmH_2_O). Three tests were done for each investigated material.

#### 2.3.6. Measurement of Water Repellency

Surface wettability was evaluated through the Spray tester, manufactured by C&B Tessile SrL, in compliance with standard EN 24920. A piece of fabric was fixed on a circular support (about 200 mm in diameter and inclination of 45°) with a metallic ring. An amount of distilled water (250 mL) was poured into a container with multi-hole end, mounted at height of about 150 mm from the disk. From openings, the liquid flowed as rain for 25–30 s on fabric surface. The final appearance of wetted fabric was compared with reference pictures associated with ISO indexes on EN standard. The final ISO index of each material was calculated by an average of three performed tests.

#### 2.3.7. Scanning Electron Microscopy

Microscopic analyses were conducted on the surface of developed specimens using a field emission scanning electron microscope (SEM, Mod. TM 3000 Hitachi, Chiyoda, Japan) under high vacuum conditions. Before testing, specimens were previously metalized with gold sputtering target.

## 3. Results and Discussion

### 3.1. Mass Per Unit of Area and the Final Appearance

**Figure 4:**
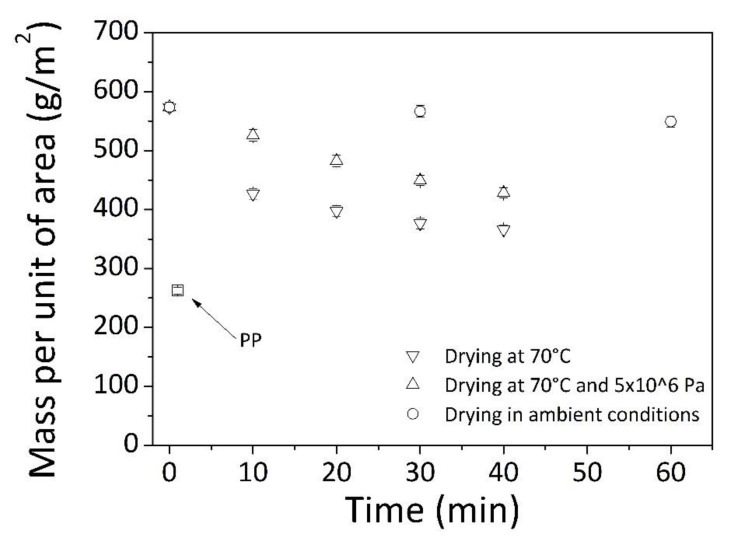
Mass per unit of area of impregnated textiles against time during drying phase.

During drying, the sample weight was constantly monitored. Results were presented in Figure 4 by showing the change of mass per unit of area as a function of time, in correspondence of specific drying setting: at ambient conditions, or at a temperature of 70 °C, also by applying a pressure of 5 × 10^6^ Pa.

From data, for wetted samples heated at 70 °C (triangles points down), the drying was finished within approximately 40 min. The final mass per unit of area of treated fabrics was found in good agreement with expected values on the base of starting sample weight and solid content in the dispersion.

Then, if together with elevated temperatures (=70 °C), also pressure of 5 × 10^6^ Pa was applied (triangles points upwards), drying process of 40 min was not effective to allow complete removal of water by leaving still wetted specimens. Probably, during this operation, the acting pressure on samples has determined little available spaces to escape of water and air not allowing the total evaporation of the liquid contained in dispersion. However, to extend the drying under these conditions did not seem a feasible solution as the application of high temperatures for more than 40 min led to slight deteriorating of fabric appearance.

Less than 4 h were required for getting completely dried samples at ambient condition (circle points). In principle, long residence times should ensure good polymer infiltration into the textile structure by favoring satisfying performance of final products. [24]

**Figure 5:**
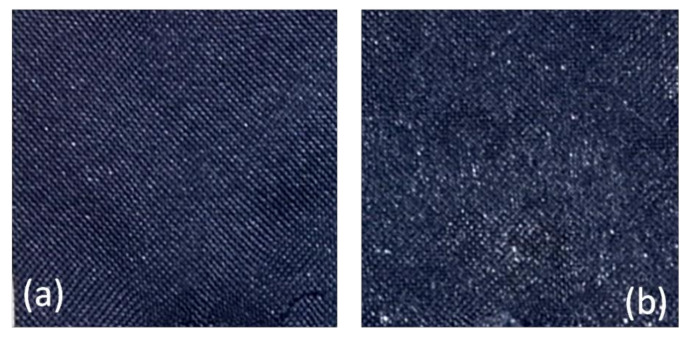
Appearance of the initial fabric (**a**) and after PUD treatment (**b**).

Following treatment, final fabric possessed a mass per unit of area equal to 367 g/m^2^ and a higher brilliance compared to pristine PP (see Figure 5).

### 3.2. Tensile Features

**Figure 6:**
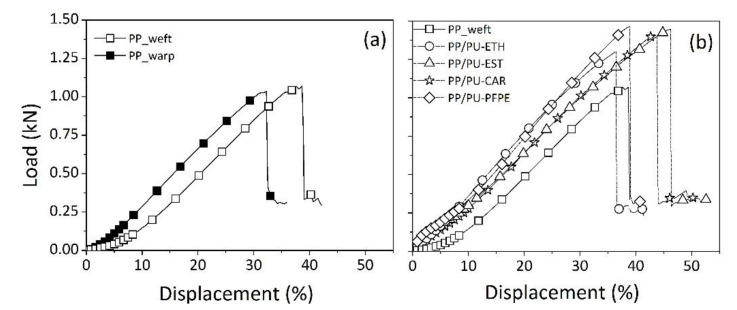
Load-displacement curves of representative samples for each tested material by tensile testing: (**a**) the basic PP fabric in warp and weft directions, (**b**) impregnated samples with different polyurethane dispersions in weft direction.

Tensile features of PP fabric, both in warp and weft direction, were displayed in Figure 6a in terms of load-displacement curves for representative samples. Average values and standard deviation of tensile breaking load have been summarized in Table 2 for all the investigated specimens.

From the data, the starting PP textile possessed a breaking load approximately equal to 1 kN, exhibiting almost isotropic characteristics in both orientations. The load-displacement curve showed a linear elastic behavior, with a change in slope particularly evident in the warp direction for tested samples in the low deformation range (< 10%). As concerning the elongation at break, average values were around 30 and 40% in the warp and weft directions, respectively.

The impregnation of PP fabric with polyurethane dispersions strongly affected the tensile properties of pristine textiles. A comparison among the typical load-displacement curves, recorded in weft direction, for each prepared sample, was reported in Figure 6b. In all cases, an improvement of the breaking load of PUD-treated specimens, compared to neat textile, was always confirmed. No strong differences appeared among the tensile features of impregnated fabrics with different types of PUD emulsions. In every case, the slope of load-displacement curve, in the range of small deformations, was about three times higher respect to untreated materials. This aspect could be intended as improvement of fabric stiffness, provided by polyurethane hardening in the textile structure. In fact, as occurred for coated or laminated fabrics [25], the polymeric treatment filled the space among filaments by bonding threads of weft and warp and producing a stiff fabric after coating.

The mechanical behavior under uniaxial stress was described by a linear trend with a change in slope for each impregnated sample in Figure 6b. Initially, PU polymer and PP fabric were stretched together; then, in correspondence of very small deformations (about 3%), in the central internal part, the polymeric PU bonds among PP filaments yielded sooner than those in the outer layer of fabric. This condition could be reasonably assumed by considering that in coated materials, filaments of inside part were lesser impregnated compared to those present in external surface. [24]. Following, the fabric continued to be stretched until the most filaments failed under tension [26].

Finally, a comparison among the breaking load of PUD-impregnated samples dried in different ways is presented in Table 2. In all cases, the maximum load remained around values of 1.39 kN by indicating no effects of drying conditions on tensile characteristics of developed products.

### 3.3. Tear Resistance

Tear strength was summarized in Table 2 in terms of average values and standard deviation for all tested specimens. In the case of PP fabric, a greater tear resistance was measured along warp (~97 N) compared to weft (~76 N) direction. 

Then, tear characteristics of PUD-treated specimens were evaluated along weft, also in the case of dried samples in different conditions. From data, it was concluded that polyurethane application did not allow benefits in tear strength. On the contrary, a small reduction in tear features seemed to be attested, in particular for treated specimens with polyether-based dispersion (65 ± 3 N), or drying conditions with pressure and temperature higher than ambient (70 ± 2 N). This result was not surprising at all, considering that the greater the crossover points among yarns, the lower the mobility of filaments and tear strength. [27]

In general, a combination of parameters affects tear features of textiles: basic fabric, weave construction, and adhesion for coated samples. To ensure high values of tear strength in fabrics, filaments should not remain fixed in one position, but they should be able to slip and move within weaving structure. Strong coating adhesion prevents the yarns motion by limiting the tear resistance [28].

In our case, being same fabric and weave construction, the only difference among investigated samples was represented by textile treatment with aqueous dispersions. So, it was accomplished that PU polymer applied to neat fabric led to an adhesion of filaments that reduced sliding, mobility and deformation by limiting the mechanical features of overall structure against tear stress.

**Figure 7:**
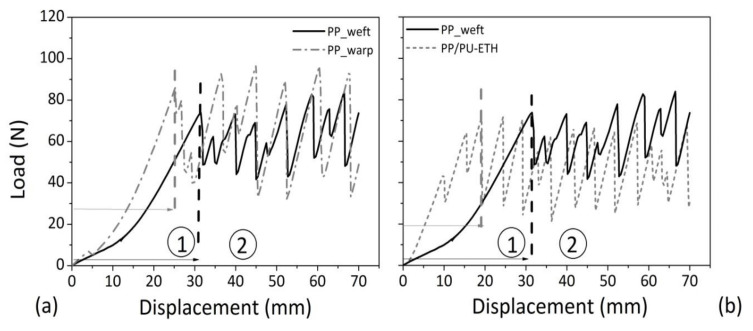
Load-displacement curves of representative samples for each tested material by tear testing: (**a**) the basic PP fabric in warp and weft direction, (**b**) impregnated samples with polyether-based polyurethane in weft direction.

Typical load-displacement curves by tear testing for neat fabric, in weft and warp direction, were observed in Figure 7a. A comparison between neat and impregnated PP with PU-ETH in the weft direction is displayed in Figure 7b.

Generally, two regions are distinguished for curves recorded during tear testing. The first part corresponds to the required energy for uniaxial deformation and first breaking in the so-called delta zone, i.e., the area of incision when longitudinal and transversal tongues are stretched under an applied tensile stress. The second zone is represented by the necessary energy for continuing the tear propagation. It consists of alternating maximum and minimum load points, derived from the axial deformation of filaments until maximum load, and the final breaking of transverse filaments in the delta point [27].

Taking into account these considerations, in Figure 7a, it was noted that in correspondence of first breaking point, the starting PP textile was endowed with a higher extensibility, but achieved for lower load values, in the weft direction (solid line) respect to warp (dashed line). Then, by comparing PP/PU-ETH and PP samples, it appeared that the PU treatment reduced initial elongation and breaking load of neat material in delta point. In fact, for PP/PU-ETH sample, the peak was achieved in correspondence of lower deformations respect to PP.

### 3.4. Abrasion Resistance

The abrasion resistance of impregnated fabrics is summarized in Table 2, in terms of minimum cycles to deteriorate the fabric appearance, and maximum cycles to rupture and perforate the sample. For neat PP, evident signs of esthetic decline appeared after 5000 abrasion cycles, attributed to pilling formation and mesh enlargement. By continuing the rubbing, the sample was broken for the cycles’ number equal to 25,000. This value was considered the abrasion resistance of starting material. 

**Figure 8:**
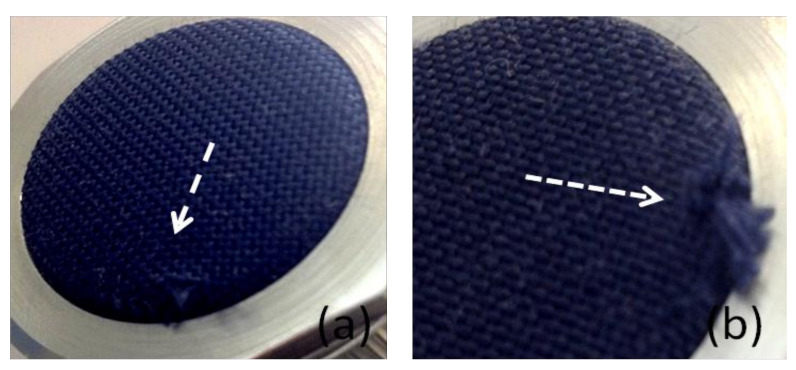
Sample deterioration during the Martindale test for the untreated PP, represented by pilling (**a**) and yarns breakage (**b**).

In Figure 8, changes in material appearance for basic textiles were shown as pilling formation (Figure 8a) and final breakage (Figure 8b). The textile treatment by polyurethane dispersions was very effective in improving the resistance against erosion of basic PP fabric. In fact, for PUD-based specimens, the tests were stopped at 100,000 cycles, and at that time the treated fabrics did not show any signs of breaking. In the case of soft-polyurethane binder (PP/PU-ETH samples), lower performance was recorded compared to other dispersions. However, the PU-ETH application represented a protection for fabric, given the increment in minimum and maximum abrasion cycles for respective samples compared to neat material.

### 3.5. Water Resistance

**Figure 9:**
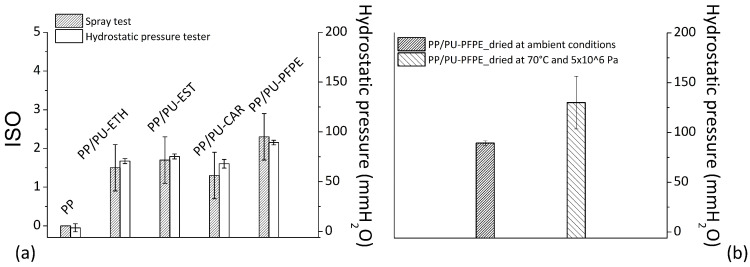
ISO index and hydrostatic pressure value for dried specimens at ambient conditions (**a**), and the effect of drying on hydrostatic pressure for PP/PU-PFPE (**b**).

Water resistance results, by ISO index and hydrostatic pressure measurements, are reported in Figure 9a for basic PP and respective impregnated specimens with different types of aqueous polyurethane.

For neat fabric, the ISO index was equal to zero and hydrostatic pressure lower than 10 mmH_2_O. Therefore, even if the polypropylene is intrinsically hydrophobic, and characterized by low surface energy [29], it does not prevent water absorption. The starting textile was completely wetted with surface exposed to the entrance of water. When the polyurethane was applied to PP-based fabrics, an increase of overall water resistance was verified in corresponding samples. In particular, the average ISO index became equal to 2.5 for PP/PU-PFPE system and about 1.5 for all other studied cases. Beyond gaining in surface wettability, the presence of polyurethane partially prevented the water transition throughout the weaving structure. In fact, by comparing neat and treated specimens, the hydrostatic pressure increased from values lower than 10 up to approximately 70 mmH_2_O. The highest augment of hydrostatic pressure (= 90 mmH_2_O) was displayed for fabrics with polyurethane containing fluoropolymer chains.

In view of these data, it can be concluded that the dispersion realized with perfluoropolyether (PFPE) backbone provided the fabric with a higher water resistance compared to other adopted polyurethanes. Probably the presence of fluoro species in PU polymer determined a reduced surface energy by making the fibers surface more hydrophobic. In this condition, the drops of water fall down from the fabric surface rather than diffuse inside it [30].

A comparison between the hydrostatic pressure of PP/PU-PFPE samples dried in ambient conditions, or under temperature of 70 °C and pressure of 5 × 10^6^ Pa was shown in Figure 9b.

From the plot, a positive outcome resulting from drying at a temperature and pressure higher than ambient can be noted. The hydrostatic pressure rose from average values of 90 mmH_2_O to 130 mmH_2_O, if superior pressure and temperature than ambient were exerted during water removal of wetted samples. Probably, in these conditions, the gap and space among filaments were closed, by creating an additional hindrance to passage of water molecules through the structure.

### 3.6. SEM Micrographs

**Figure 10:**
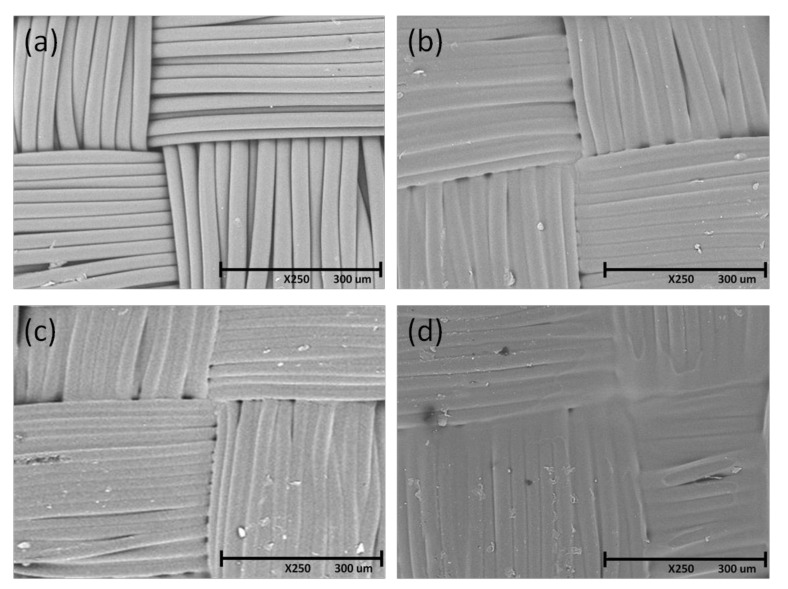
SEM micrographs of the sample surfaces for neat PP (**a**), and PP/PU-PFPE dried at ambient conditions (**b**), dried at 70 °C (**c**), or at 70 °C and 5 × 10^6^ Pa (**d**).

The influence of drying conditions on the physical distribution of PU polymer inside the textile weaving was described through SEM analyses. Imagines at an equal magnification (× 250) were reported in Figure 10 for following materials: neat PP (a), impregnated PP with PU-PFPE dried at ambient condition (b), at 70 °C (c), or at 5 × 10^6^ Pa and 70 °C (d).

For starting fabric (Figure 10a), filaments appeared well-defined, intertwined according to weft and warp direction, with distinct edges, separated with each other. Due to PU application (Figure 10b) and drying at ambient, the fabric surface looked like more opaque with boundary lines among yarns less evident and distinguishable compared to neat PP. This appearance was considered a direct consequence of bonding effect of PU polymer among fibers.

By comparing treated textiles in the case of drying at 70 °C (Figure 10c) or room temperature (Figure 10b), the sample surface seemed to be fuzzier in the first case than in the second one. This was considered an effect of higher temperatures than ambient, applied during drying of impregnated fabric. In this condition, a strictly and fast removal of water took place for polymeric treatment. 

Other considerations were done when the drying was conducted at higher temperature and pressure than ambient (Figure 10d). In latter situation, the center of image seemed to be very opaque but structure around edge seemed to be detectable. The identity of filaments was reduced, the texture weave was loosed, more compacted body with flattened constituents to each other seemed to be realized. This effect was attributed to pressure and temperature higher than ambient, contemporary acting during the drying process: while the first parameter has crushed the sample, the second one has fixed the shape.

To quantitatively underline differences in final appearance of prepared samples at different drying conditions, SEM pictures were reworked by ImageJ software.

The pixel gray value distribution in plain-image was shown by plotting the number of pixels for specific intensity (vertical axis) vs brightness (horizontal axis). Intensity changes in terms of mean, median and modal intensity, were calculated for each representative histogram of specific samples.

**Figure 11:**
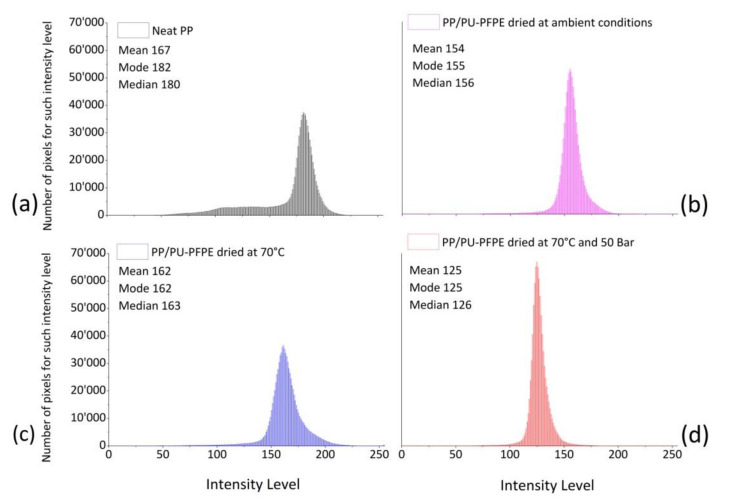
Histograms of gray imagines by SEM micrographs for neat PP (**a**), and PP/PU-PFPE dried at ambient conditions (**b**), dried at 70 °C (**c**), or at 70 °C and 5 × 10^6^ Pa (**d**).

PP surface is represented in Figure 11a by a wide distribution of pixel numbers by covering a greater range of intensity level compared to the other samples. The smaller mean (167) respect than median (180) confirmed low scores constituting the long tails moved to the left. 

For PUD-impregnated specimens, mode, median and mean values were similar in number among themselves by attesting a symmetrical distribution in gray intensity. 

However, brightness achieved in treated materials for drying at 70 °C (median of 163) (Figure 11c) was larger than that for drying at ambient conditions (median of 156) (Figure 11b) and by applying higher pressure and temperature than the room (median of 126) (Figure 11d). So, drying at 70 °C led to less opaque and bright surface; whereas, pressure higher than ambient, in addition to a temperature of 70 °C, produced dark and less defined parts. In the latter case, the narrowest and most symmetrical distribution of pixels number associated with the highest ordinate value was measured.

## 4. Discussion

In this work the effect of polyurethane impregnation on final performance of commercial polypropylene-based textiles was presented. During the impregnation process the following steps could be reasonably hypothesized: given the high wettability of fabric (as confirmed in water resistance testing), good permeability of dispersion could be imagined with easy infiltration in textile structure. The polyurethane was present in continuous aqueous medium in form of micelle aggregates (about in size of 20–200 nm). It possessed a high surface energy and began to coalesce as water evaporated [31]. Firstly, PU polymer saturated the surface structure by filling the voids outside the yarns (macro-flow), then, it crossed transversely and penetrated into bundles (micro-flow) [24].

Experimental results proved that PU impregnation treatment of fabric significantly affected the mechanical behavior and resistance against water of developed materials. In the face of weight gain, no significant difference was found in aesthetics of PUD impregnated fabric compared to neat PP. Thanks to protective polymeric support, fibers and filaments of basic material were preserved, and made globally strong. According to work of Masteikaite and Saceviciene [25], common coating and lamination methods determined an increase in stiffness of treated textiles since the applied material joined together weft and warp threads. In this way, more filaments participated in sharing the tensile load by leading to an increase of mechanical strength. Also in the case of impregnation, a similar outcome was expected. In fact, as shown in SEM micrographs, for neat fabric (Figure 10a), filaments appeared distinct from each other. Then, in presence of polyurethane (Figure 10b), a thin opaque layer seemed to be deposited homogeneously on fabric surface, making edges of filaments linked among themselves. These considerations were analogously confirmed in a previous work on polyester-based fabrics [5].

Same positive aspects, determinant for increasing the resistance against tension, were proven detrimental for tear characteristics. In fact, by analyzing structural parameters affecting tear strength, Eltahan [28] concluded that the coating adhesion was responsible of hardening in structure, but it hindered mobility and sliding of yarns by reducing the strength against tear stress. Also in our case, it was confirmed that the bonding effect between fibers in neat fabric due to PU treatment, and specific drying conditions (pressure and temperature higher than ambient), led to a loss of tear strength.

As concerning abrasion resistance, remarkable performance was demonstrated when the polyurethane was applied to fabric surface. Abrasion is a complex phenomenon, often related to an erosion process of material surface by leading to matter removal with surface deterioration until breakage. In the case of textiles, various aspects, such as fibre, yarn, fabric properties and finishing processes, play a determining role in affecting the surface and abrasion mechanism [32]. During testing, the deformation remained constant, and was not considered responsible for sample breakage [33]. On the contrary, the rubbing and scraping action on surface has produced a weakening of material, loss in mechanical resistance, and worsening in outward aspect [34]. When the polyurethane was deposited inside the textile weaving, it contributed to protect against the wearing, and determined an increase of fabric durability. This effect was attributed to the creation of smooth and less corrugated surfaces (as shown in SEM micrographs) that restricted fiction during contact with abrading medium [35]. So, due to polyurethane infiltration, the overall structure became more resistance and suitable to hold up superior mechanical stress. At regards, also in work of Akaydin [36], it was established that the higher the yarns compactness the higher the abrasion resistance and the lower the pilling formation. The result was ascribed to a reduced hairiness, and increased structural strength by more participating yarns in sustaining the resistance of material.

Finally, for fully outlining effects of PU impregnation on PP fabric, the water resistance of developed PUD materials was analyzed. In the framework of the textile field, two terms sre used to define the water resistance: “water repellent” and “waterproof”. Although with essentially different meaning, these adjectives are often confused and utilized improperly. Usually, textiles are endowed with waterproofness when pores and voids in the structure are filled by substances that prevent the passage of air and water. On the contrary, it could be ascribed characteristics of water repellent to fabrics, when fibers are made of hydrophobic constituents, but pores and openings of weaving remain accessible [37]. In the examined case, even if the polypropylene was chosen as constituent fabric material, the textile surface was always completely wettable. To increase the water resistance, both in terms of surface wettability and water passage through fabric, it was considered essential to close the empty spaces among filaments of weft and warp, by which liquid infiltrated and crossed fabric thickness. In this task, the polymer impregnation resulted to be effective, since it allowed to bond threads, fill blanks and prevent the water entrance. This effect was enhanced by applying elevated pressure and temperature than the room during drying phase. In these conditions, the flattening of fabric resulted in an increased bond among yarns, as shown by SEM images (Figure 10d). Higher pressure and temperature than ambient during drying were not considered a useful operational setting, in view of the worsening of tear performance and final aesthetics.

## 5. Conclusions

PP-based-textiles were treated by an impregnation method with four selected commercial-available waterborne polyurethanes, differing in their chemistry. Characteristics of tensile, tear, abrasion, and water resistance were analyzed for the prepared samples. The effect of drying parameters on desired features of treated fabrics was also investigated. Experimental results highlighted different benefits coming from polyurethane application to the textile weave of neat fabric. The impregnation treatment did not alter the appearance of pristine material, despite an increase in rigidity and weight. In all PUD-treated samples, an increase in breaking load, abrasion resistance, water repellency and waterproofness was always detected with respect to untreated specimens. In terms of tear features, there weren’t noticeable differences between PU-treated and untreated specimens. The presence of PU polymer attached to the fabric rather led to a worsening of tear strength. By comparing the effect of different aqueous PU solutions on performance of final products, no substantial variations were verified among all tested features. Yet, once applied, the polyurethane realized with perfluoropolyether species provided the fabrics of a higher water repellency compared to other PUD products. Finally, drying conditions performed at elevated temperature and pressure than room conditions didn’t affect tensile features, reduced tear strength, and increased hydrostatic water pressure of PUD-based samples.

## Figures and Tables

**Table 1:** Technical datasheet of the chosen commercial dispersions.

Sample Name	PU-ETH	PU-EST	PU-CAR	PU-PFPE
Nature of Dispersion	Anionic	Anionic	Anionic	Anionic
Type of Polyurethane	Aliphatic polyether	Aliphatic polyester	Aliphatic polycarbonate	Perfluoropolyether (PFPE) backbone-containing
Solid Content (%)	34	35	35	25

**Table 2:** Mechanical performance of investigated samples.

Sample	Tensile Load (N)	Tear Strength(N)	Abrasion Resistance (Cycles)	
			Minimum	Maximum
Basic Fabric PP				
weft	1.07 ± 0.03	76 ± 5	> 5000	25,000
warp	1.03 ± 0.05	91 ± 3		
*(a) Effect of the Different PUD Dispersions*				
PP/PU-ETH weft			> 10,000	95,000
weft	1.30 ± 0.21	65 ± 3		
PP/PU-EST			10,000	> 100,000
weft	1.46 ± 0.13	70 ± 4		
PP/PU-CAR			10,000	> 100,000
weft	1.44 ± 0.35	72 ± 5		
PP/PU-PFPE			10,000	> 100,000
weft	1.42 ± 0.17	73 ± 7		
*(b) Effect of Drying Conditions*				
PP/PU-EST_Drying at 70 °C			/	/
weft	1.46 ± 0.15	69 ± 5		
PP/PU-EST_Drying at 70 °C and 5 × 10^6^ Pa			/	/
weft	1.32 ± 0.19	70 ± 2		

## Data Availability

The data presented in this study are available on request from the corresponding author.
